# Erythropoietin for the prevention of postoperative neurocognitive disorder in older adult patients undergoing total joint arthroplasty: a randomized controlled study

**DOI:** 10.1186/s12871-024-02770-9

**Published:** 2024-11-15

**Authors:** Eun Jung Kim, Kwan Kyu Park, Su Youn Choi, Hyang Mi Ju, Tae Lim Kim, Jeongmin Kim, Soo Yeon Kim, Bon-Nyeo Koo

**Affiliations:** 1 Department of Anesthesiology and Pain Medicine, Yonsei University College of Medicine, Seoul, Republic of Korea; 2 Anesthesia and Pain Research Institute, Yonsei University College of Medicine, Seoul, Republic of Korea; 3 Department of Orthopedic Surgery, Severance Hospital, Yonsei University College of Medicine, Seoul, Republic of Korea; 4 Department of Anesthesiology and Pain Medicine, Yonsei University College of Medicine, Yongin Severance Hospital, Yongin-si, Republic of Korea

**Keywords:** Erythropoietin, Postoperative neurocognition, Postoperative delirium

## Abstract

**Background:**

Post-operative delirium (PD) is a common post-operative complication with significant clinical and financial impacts on patients. Erythropoietin (EPO), a multi-functional glycoprotein hormone, exhibits erythropoietic and non-erythropoietic anti-inflammatory properties. This study aimed to determine the role of perioperative EPO administration in the development of postoperative delirium in older adult patients undergoing total joint arthroplasty.

**Methods:**

Seventy-one patients (> 65 years old) scheduled for total joint arthroplasty were randomly assigned to two groups: EPO-treated (EPO, *n* = 35) and placebo (control, *n* = 36). All patients completed the Mini Mental State Examination (MMSE) pre-operatively and on post-operative day (POD) 2. The confusion assessment method (CAM) was used to assess the patients until discharge (POD 5). Serum C-reactive protein (CRP) and inflammatory cytokine levels were measured and compared pre- and post-operatively. The development of delirium and cognitive dysfunction was evaluated post-operatively.

**Results:**

One patient in the control group developed delirium on POD 2 (3.2%), whereas no patient in the EPO group developed PD (0% vs. 3.2%, *p* = 0.500). Post-operatively there was no significant difference in MMSE scores between groups. Both groups showed increases in pro- and anti-inflammatory cytokine levels, with no significant differences. Similarly, CRP levels, neutrophil/lymphocyte ratio (NLR), and platelet/lymphocyte ratio (PLR) showed no intergroup differences in post-operative inflammatory responses.

**Conclusions:**

Perioperative EPO reduced the incidence of post-operative delirium, although not statistically significant, with no differences in post-operative cognitive function and inflammatory responses.

**Trial registration:**

The trial was registered on December 12, 2023 at http//clinicaltrials.gov, registration number NCT06178835.

## Background

Post-operative delirium (PD) is a clinical syndrome defined as an acute and fluctuating alteration in consciousness levels, along with disorganized thinking and an altered attention span occurring after surgery [1]. The incidence of PD varies widely among study populations with it affecting up to 60% of vulnerable populations [2]. Risk factors for PD include prolonged transfusion time [3], release of pro-inflammatory factors due to exposure to extracorporeal circulation [4], and surgical stress, along with frequently cited predisposing factors such as old age, impaired sensory and cognitive function, and coexisting medical conditions [5, 6].

Post-operative cognitive dysfunction is a multifactorial disease entity with precise pathogenesis that is unidentified. However, considerable attention has been paid to the role of neuroinflammation and immune activation following surgery and anesthesia during its development [7, 8]. Post-operative neuroinflammation involves a systemic inflammatory response which could be induced by peripheral inflammation and is possibly triggered during the perioperative phase. Evidence suggests that neuroinflammatory changes, characterized by the release of pro-inflammatory mediators, contribute to detrimental structural and functional alterations within the brain, including compromised blood-brain barrier integrity and monocyte-derived macrophage migration into the brain parenchyma [9–11].

Erythropoietin (EPO), a multi-functional glycoprotein hormone primarily known to stimulate erythropoiesis, has also been recognized for its neuroprotective properties and potential for cognitive enhancement [12–15]. Animal studies have demonstrated EPO’s ability to improve spatial learning and memory and to reduce neuroinflammatory reactions and oxidative stress markers [13, 16, 17]. However, concerns about side effects such as thromboembolism, hypertension, and even tumour progression [18–20] have limited human studies investigating the cognitive effects of EPO and its clinical use.

This study aimed to assess the impact of perioperative EPO administration on the incidence of postoperative delirium and further investigate its effects on post-operative cognitive function and inflammatory response in older patients undergoing total joint arthroplasty.

## Materials and methods

After receiving institutional review board approval, the study was conducted at Severance Hospital, Yonsei University Health System and was analyzed in accordance with Consolidated Standards of Reporting Trials (CONSORT) guidelines. Patients aged 65 years and older scheduled for elective total knee or hip arthroplasty surgery were screened for eligibility. The exclusion criteria included altered consciousness, underlying neurological conditions such as delirium and dementia, and an inability to comprehend or provide consent. Participants were randomly assigned 1:1 to either the EPO-treated or placebo group using a computer-generated table created by an investigator not involved in the neurological assessment. The Mini-Mental State Examination (K-MMSE II) was administered pre-operatively and two days post-operatively by a physician blinded to the patient and treatment information to confirm changes in postoperative cognitive function. The Confusion Assessment Method (CAM) was used twice daily for five days post-operatively to assess the occurrence of post-operative delirium [21]. All tests were validated previously and the copyright version of MMSE-2 in the Target Language (Korean) within the Territory (Republic of Korea) was used with the license acquired from Psychological Assessment Resources, Inc (www.parinc.com).

### Perioperative Management

Experienced physicians performed anesthesia and analgesia management. Patients underwent either spinal or general anesthesia. Spinal anesthesia involved the administration of hyperbaric bupivacaine (10–12 mg) supplemented with intravenous propofol or midazolam for intraoperative sedation. General anesthesia was induced with propofol (1-1.5 mg/kg), rocuronium bromide (0.6 mg/kg), and remifentanil (0.05 mg/kg/min), followed by maintenance with sevoflurane at an end-tidal concentration of 0.4–1.5% (within age-corrected 0.5–1.5 minimum alveolar concentration) in an oxygen-air mixture, along with remifentanil infusion.

Each patient received either 500 IU/kg of recombinant human erythropoietin (rhEPO) dissolved in 2 ml of saline or an equivalent volume of normal saline intravenously three times: on the day before surgery, at the start of surgery, and on post-operative day 1 (POD 1). For postoperative analgesia, 1 ${\mu}g$/kg bolus of fentanyl was administered immediately at the end of surgery and at the recovery room. At the ward, patients were given 650 mg of acetaminophen twice daily, and tramadol 50 mg intramuscular injection were given as a rescue analgesic. Serum levels of C-reactive protein (CRP) and inflammatory cytokines were measured twice: at baseline on the morning of surgery and post-operatively. Peripheral blood samples were collected before anesthesia induction (T0) and after surgery (T1), immediately centrifuged, and stored at -80 °C until further analysis. Plasma levels of IL-10, TNF-α, and CRP were measured using enzyme-linked immunosorbent assays (ELISA). Intraoperative fluid input, blood loss, and transfusion volume were recorded. A standard hospital protocol for total knee or hip arthroplasty was followed, and routine post-operative rehabilitation was initiated in all patients. The investigator responsible for post-operative follow-up was blinded to patient allocation.

### Postoperative Assessments

PD development was assessed until the POD 5. Surveillance, conducted by an investigator blinded to patient allocation, involved two daily visits and patient and guardian interviews using the CAM. The occurrence of PD was determined using the CAM criteria. Postoperative cognitive function testing using the MMSE was performed on POD 2.

#### Statistical analysis

The sample size was determined based on the previously reported incidence of post-operative neurocognitive dysfunction after rhEPO administration in patients undergoing coronary artery bypass graft (8.3% vs. 38.0%) [22]. To detect a 29.7% difference in incidence between groups with 80% power at a significance level of 5% (α = 0.05, power = 80%), 35 participants were required per group. To allow for a 10% dropout rate, 38 patients were enrolled in each group. Parametricity was confirmed using Shapiro–Wilk and Kolmogorov–Smirnov tests. Parametric continuous variables were analyzed using the independent *t*-test, whereas nonparametric variables were analyzed using the Mann–Whitney *U* test. Intergroup comparisons of categorical variables were conducted using the Fisher exact test or the χ² test. Propensity score matching (PSM) analysis was also performed to adjust differences in potential covariates by a 1:1 matching ratio. In the matched patient groups, continuous variables were analyzed using paired *t*-test and categorical variables were analyzed using Wilcoxon signed rank test. All statistical analyses were performed using SAS version 9.4 (SAS, Cary, NC, USA), SPSS (version 26.0; IBM, Chicago, IL, USA), and GraphPad Prism 10 (GraphPad, Boston, MA, USA). *P-*values < 0.05 were considered statistically significant.

## Results

### Patient Demographics

**Fig. 1:**
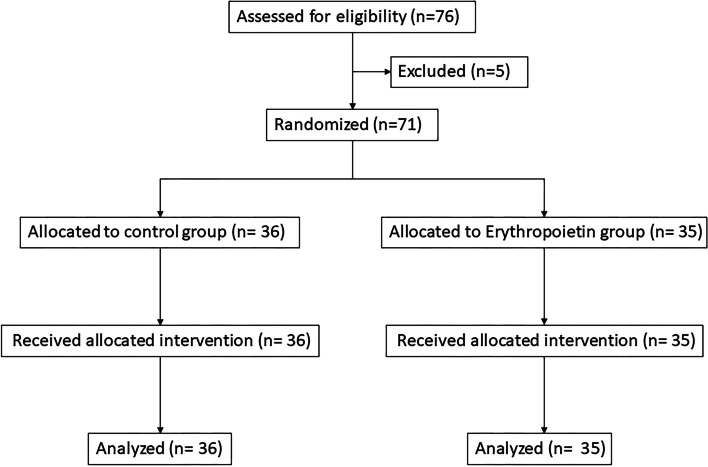
CONSORT flow diagram of the study protocol

A CONSORT flow diagram summarizing the study protocol is shown in Fig. 1. Of the 76 enrolled patients from September 2017 to July 2019, only 71 were included in the analysis (control group: 36; EPO group: 35), five patients were excluded due to missing data. The basic demographic and clinical characteristics of patients in both groups are shown in Table 1. No significant differences were observed in patient’s age, sex, body mass index, or underlying medical comorbidities between the treatment groups. Perioperative patient data, including surgery time (EPO, 106.54 ± 7.55 min, Control, 95.31 ± 7.55 min, *P* = 0.304), anesthesia time (EPO, 140.86 ± 8.29 min, Control, 129.36 ± 8.5 min, *P* = 0.337), and fluid balance during surgery (e.g., amount of intraoperative fluid intake, urine output, blood loss (EPO, 100.0 mL (IQR 90.0-100.0), Control, 100.0 mL (IQR 100.0-162.5), *P* = 0.371), and transfusion volume), were comparable between the groups. After performing the PSM analysis, 62 patients were included. The baseline characteristics between the two groups were listed with all adjusted variables that were comparable after PSM (Table 1).

**Table 1:** Patient Demographics in two groups before and after PSM

Variables	Before Matching			After Matching			
	EPO group (*n* = 35)	Control group (*n* = 36)	SMD	EPO group (*n* = 31)	Control group (*n* = 31)	SMD	*P*-value
Age (years)	72.1 ± 0.9	71.5 ± 1.0	0.107	72.1 ± 0.9	71.9 ± 1.0	0.025	0.533
Male sex	6 (17)	6 (17)	-0.012	5 (16)	5 (16)	0.000	0.655
BMI (kg/m^2^)	25.79 ± 0.81	26.27 ± 0.46	-0.100	25.77 ± 0.91	26.12 ± 0.51	-0.074	0.645
Hypertension	22 (63)	20 (56)	0.149	18 (58)	19 (61)	-0.066	0.796
Diabetes	8 (23)	7 (19)	0.080	6 (19)	7 (23)	-0.076	0.480
Coronary artery disease	1 (3)	0 (0)	0.169	0	0	0.000	0.317
Chronic obstructive pulmonary disease	1 (3)	1 (3)	0.005	1 (3)	1 (3)	0.000	0.317
Types of Anesthesia							
General Anesthesia	20 (57)	11 (31)	0.530	16 (52)	11 (35)	0.321	0.317

Data are expressed as mean ± standard error (SE), or number of patients (percentage)

*PSM* Propensity score matching, *EPO* Erythropoietin, *SMD* Standardized Mean Difference, *BMI* Body mass index

### Incidence of Postoperative Delirium and MMSE scores

None of the patients in the EPO group experienced delirium, whereas one patient in the control group developed delirium (0 vs. 3.2%, *P* = 0.500). Both groups exhibited a decrease in MMSE scores after surgery, with a smaller decrease observed in the EPO group than in the control group on postoperative day 2, although statistical significance was lacking [0 (0–1) vs. 1 (0–1), *P* = 0.825] (Table 2). None of the patients developed any EPO-related severe side effects, such as cerebral hemorrhage, edema, or thromboembolic events.

**Table 2:** Postoperative cognitive function in two groups before and after PSM

	Before Matching		After Matching		
	EPO (*n* = 35)	Control (*n* = 36)	EPO (*n* = 31)	Control (*n* = 31)	*P*-value
MMSE Preop	27.54 ± 1.93	27.06 ± 1.66	27.45 ± 0.36	27.23 ± 0.28	0.470
MMSE POD 2	27.31 ± 1.80	26.36 ± 1.71	27.16 ± 0.33	26.61 ± 0.27	0.371
ΔMMSE	0 (0–1)	1 (0–1)	0 (0–1)	1 (0–1)	0.825

Data are expressed as mean ± SE, or median (Q1, Q3)

*PSM* Propensity score matching, *EPO* Erythropoietin, *MMSE* Mini-mental state examination, *POD* Postoperative day

### Routine Laboratory Tests and Inflammatory Cytokines

**Fig. 2:**
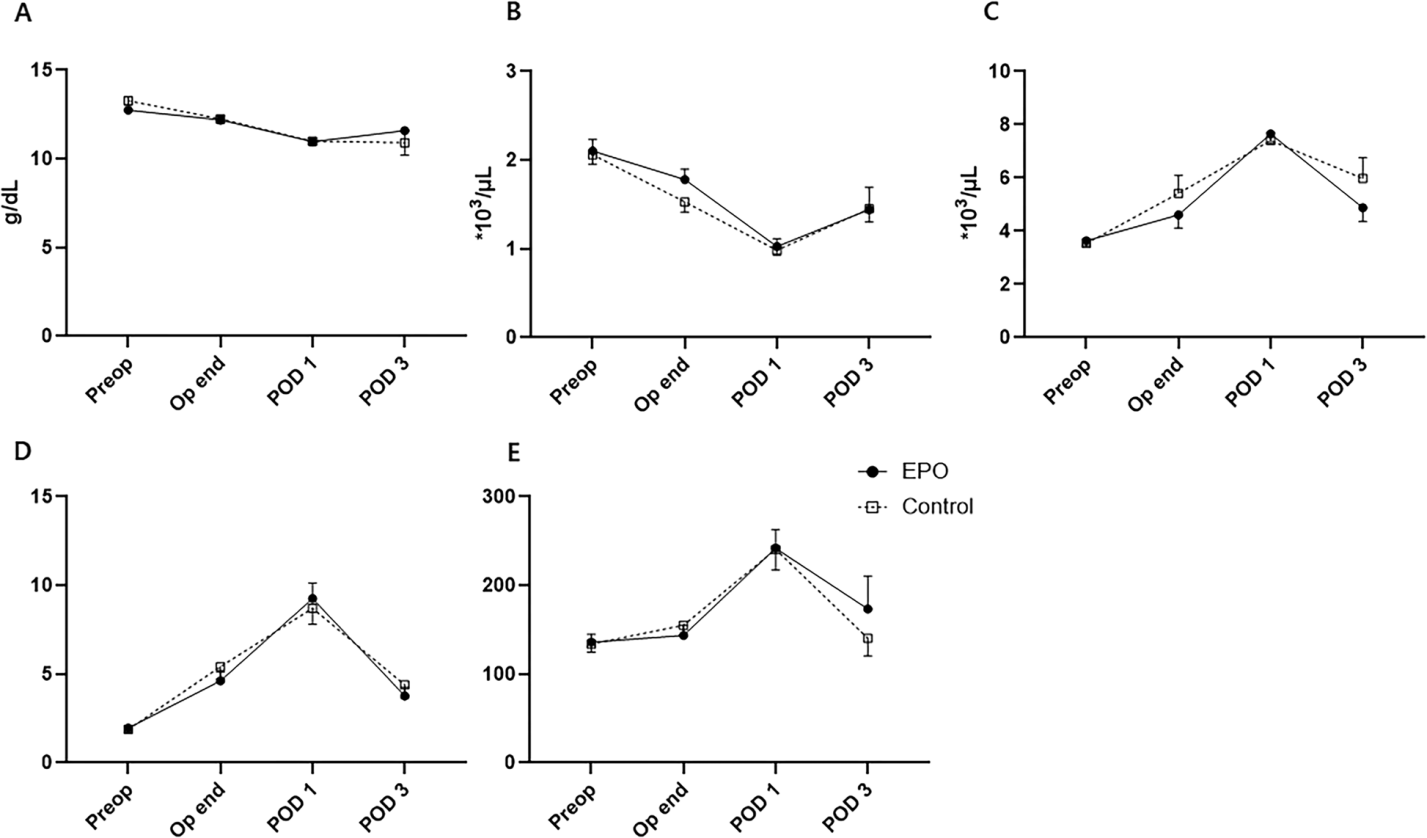
Intraoperative changes in laboratory tests in older adult patients undergoing total joint arthroplasty (**A**) Hemoglobin, (**B**) lymphocyte, (**C**) neutrophil, (**D**) neutrophil/lymphocyte ratio, (**E**) platelet/lymphocyte ratio in all patients. EPO, erythropoietin; POD, postoperative day. The whiskers represent the standard error

**Fig. 3:**
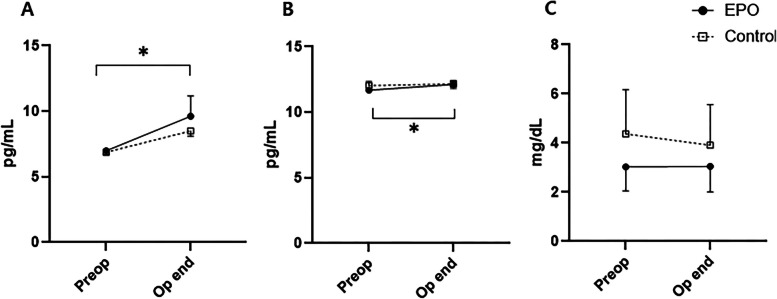
Intraoperative changes in serum inflammatory cytokines and C-reactive protein (**A**) IL-10, (**B**) TNF-α, (**C**) CRP in all patients. EPO, erythropoietin. The whiskers represent the standard error. *p-value of < 0.05, compared with the value obtained preoperatively within the EPO group

Changes in routine laboratory tests, including serum hemoglobin (Hb) levels, were not significantly different among the groups over time (Fig. 2). Other biomarkers related to systemic inflammation, such as circulating lymphocyte, neutrophils count, neutrophil-to-lymphocyte ratio (NLR), and platelet-to-lymphocyte ratio (PLR), showed similar trends, with no significant differences between the two groups (Fig. 2). Serum levels of inflammatory cytokines and CRP pre- and post-surgery are shown in Fig. 3. The levels of anti-inflammatory (IL-10) and pro-inflammatory (TNF-α) cytokines increased in both groups after surgery, with a significant increase noted in the EPO group. However, no significant intergroup differences were observed.

## Discussion

Based on our results, EPO reduced the incidence of postoperative delirium, although it was not statistically significant. There were no differences in postoperative cognitive function and inflammatory responses in older patients undergoing total joint arthroplasty.

EPO has emerged as a promising candidate with anti-inflammatory properties, as demonstrated in preclinical and clinical studies, particularly for mitigating neuroinflammatory responses associated with neurocognitive function. This study aimed to elucidate the protective effects of EPO against PD, potentially induced by inflammatory responses following surgery and anesthesia, from the perspective of attenuating neuroinflammatory responses.

EPO is a hematopoietic growth factor that stimulates erythropoiesis, with known clinical efficacy in promoting hematopoiesis and exerting systemic anti-inflammatory effects [23–25]. However, in addition to its classic hematopoietic role, EPO has demonstrated effectiveness in exerting anti-inflammatory effects in various clinical fields and subjects. Suarez-Mendez et al. described a significant decrease in serum levels of pro-inflammatory cytokines, including IL-6 and TNF-α, following EPO treatment in patients with diabetic neuropathy [26]. Similarly, Tanaka et al. revealed that using erythropoietin-stimulating agents was significantly correlated with reduced levels of CRP, a key marker of inflammation and cardiovascular disease, in patients with chronic kidney disease [27]. These findings underscore the beneficial effects of EPO on systemic inflammation, highlighting its potential as a therapeutic agent for various clinical conditions.

EPO induces neuroprotection in the central nervous system (CNS) by interacting with the EPO receptors (EpoRs), primarily expressed by neurons. Exogenous EPO also induces EpoR and EPO expression, thereby enhancing neuroprotection. EPO activates Protein Inositol 3 kinases (PI3K)/protein kinase B (PKB, also known as Akt), Janus Kinase/signal transducer and activator of transcription (JAK/STAT), and mitogen-activated protein kinase (MAPK) pathways [28, 29]. These pathways mediate various protective mechanisms in the CNS, including neurite outgrowth, anti-apoptosis of neurons and microvascular endothelial cells, and remyelination [30–32]. EPO also promotes vascular endothelial growth factor expression, angiogenesis, and neuroprotection.

Macrophages are specialized immune cells primarily function as phagocytes engulfing and clearing cellular debris, pathogens, and apoptotic cells. Macrophages also release pro-inflammatory cytokines and chemokines to amplify the immune response and recruit other immune cells to sites of inflammation. The neuroprotective mechanism of EPO involves its interaction with macrophages, which are key mediators of innate immunity and exhibit either a pro-inflammatory M1 phenotype or an anti-inflammatory M2 phenotype. Our previous study showed that this mechanism of EPO is manifested by suppressing the expression of M1-related genes and promoting the expression of M2-related genes post-operatively [17]. We also found that EPO decreased the proportion of macrophages/microglia expressing the M1 surface marker, CD40, and increased the expression of the M2 surface marker, CD206. This was accompanied by reduced pro-inflammatory cytokine production in the hippocampus [17, 33].

Regarding the mechanism of EPO in CNS inflammation, it is imperative to highlight the significant contribution of brain-derived neurotrophic factor (BDNF). EPO also upregulates the expression of BDNF, a crucial neurotrophin involved in neuronal survival, synaptic plasticity, and neurogenesis [34]. BDNF plays a pivotal role in regulating neuroinflammatory responses and promoting neuronal repair and regeneration. Thus, the interaction between EPO and BDNF underscores the potential therapeutic benefits of EPO in mitigating inflammation in the CNS and enhancing neuroplasticity and neuroprotection [34, 35].

Despite the beneficial effects of EPO, such as erythropoiesis and neuroprotection, its side effects require careful consideration during administration. Although the probability of occurrence is low, studies have shown a significantly increased risk of serious adverse effects of EPO, including death, intracerebral hemorrhage, brain edema, and thromboembolic events, with high doses and co-administration of tPA [36, 37]. Several studies have reported minor adverse events, such as nausea and headaches, with no significant differences in frequency between the rhEPO and control groups [38].

This study has several limitations. First, it used the lowest dose that satisfied patient safety and effectiveness. The dose we used in our study i.e., 500 IU/kg dose of EPO (three times during perioperative period, total 1500 IU/kg dose of EPO), is much lower than the EPO doses of 100,000 to 200,000 IU used in previous studies that reported adverse effects of EPO. Therefore, an insufficient amount of EPO might be responsible for no significant effect on the occurrence of post-operative delirium. Furthermore, while the administration of EPO led to a notable increase in the anti-inflammatory cytokine IL-10 post-operatively, it is noteworthy that there was also a simultaneous increase in the pro-inflammatory cytokine TNF-α, which possibly implies ambiguity due to the insufficient dosage of EPO. A second limitation is the patient selection. As the study consists of a relatively small sample size of patients belonging to a single ethnicity and admitted at a single institution, future large-scale studies are needed for results to be generalized to other patient populations as well as other types of surgery. Moreover, more detailed exclusion criteria for patient selection beyond underlying neurological conditions could have yielded clearer results, given the characteristics of older patients with various underlying medical conditions. However, despite the small sample size, our results are encouraging, showing the reductions in the incidence of post-operative delirium and the degree of decline of cognitive function two days postoperatively. Third, although PSM was adopted to reduce the selection bias, the possibility of unobserved differences between the groups cannot entirely be excluded. Finally, the absence of BDNF measurement, a key mechanism influenced by EPO, and the lack of long-term follow-up for changes in cytokines, neurocognitive assessment, and studies on neuroinflammation are a few more limitations of this study.

## Conclusions

In conclusion, EPO reduced both the incidence of post-operative delirium and the degree of decline of MMSE scores, although not statistically significant. However, future large-scale, long-term follow-up studies are necessary to confirm the effects of EPO on post-operative neurocognitive disorders.

## Data Availability

The datasets used and analyzed in the current study are available from the corresponding author on reasonable request.

## Abbreviations

PD: Post-operative delirium
EPO: Erythropoietin
CONSORT: Consolidated Standards of Reporting Trials
MMSE: Mini Mental State Examination
POD: post-operative day
CAM: confusion assessment method
CRP: C-reactive protein
NLR: neutrophil/lymphocyte ratio
PLR: platelet/lymphocyte ratio
rhEPO: recombinant human erythropoietin
ELISA: enzyme-linked immunosorbent assays
PSM: Propensity score matching
Hb: hemoglobin
CNS: central nervous system
EpoR: EPO receptor
PI3K: Protein Inositol 3 kinases
PKB: protein kinase B
JAK/STAT: Janus Kinase/signal transducer and activator of transcription
MAPK: mitogen-activated protein kinase
BDNF: brain-derived neurotrophic factor
SMD: standardized mean difference
BMI: body mass index
